# Short Communications: An improved automated colorimetric analysis of fructose in fermentation media

**DOI:** 10.1155/S1463924680000478

**Published:** 1980

**Authors:** R. B. Roy, A. Buccafuri

**Affiliations:** Technicon Industrial Systems Tarrytown New York 10591 USA


### Short Communications

### An improved automated colorimetric analysis of fructose in fermentation media

**R. B. Roy and A. Buccafuri**
*Technicon Industrial Systems, Tarrytown, New York 10591*

There is a widespread interest in the commercial application of D-glucose-isomerase for the large-scale conversion of D-glucose into fructose-containing syrups[1]. As such, there is a need for a rapid and reliable method for the routine analysis of glucose-isomerising activity in fermentation broths. The analytical methods currently in use include the incubation of the enzyme preparation with a solution of D-glucose followed by colorimetric analysis of the fructose produced [2,3].

Of the many proposed colorimetric methods such as the reaction of anthrone with sulphuric acid [4-6], resorcinol with hydrochloric acid [7], thiobarbituric with hydrochloric acid [8], and the cysteine-carbazole-sulphuric acid procedure, the latter commonly known as the "Dische Reaction" [9,10] stands out as being particularly advantageous for the selective and sensitive analysis of fructose in the presence of other carbohydrates. Cadmus and Strandburg [11] reported an automated procedure using only cysteine and sulphuric acid reagents for the quantitative assay of D-fructose in the presence of a large excess of aldoses and other ketoses. Lloyd *et al* [12] described a semi-automated procedure using the Dische reaction for the determination of D-fructose in biological materials.

This note describes a simplified and rapid automated procedure which retains the accuracy of the cysteine-carbazole-sulphuric acid procedure while using milder reaction conditions than those formerly prescribed [12-14]. There are also fewer analytical modules included than previously described. The method is capable of analysing fructose in the range of 0.01–0.1 g/l solution. Twenty samples per hour are analysed.

### Materials and method

All chemicals should be of reagent grade. Distilled water is used throughout to make up the solutions to volume.

### Renex-30 solution, 30% (w/v)

Weigh 30.0 g Renex-30 (available from J.T. Baker Chemical Company, Phillipsburg, New Jersey 08865 USA) into a 100 ml volumetric flask. Make up to volume with water. Store the reagent in a plastic bottle.

### L-cystein hydrochloride solution, 2% (w/v)

Weigh 2.0 g L-cysteine hydrochloride into a 100 ml volumetric flask. Make up to volume with water. Add 0.5 ml Renex-30 solution and mix the contents thoroughly. Keep the reagent in a brown bottle and prepare fresh daily.

### Carbazole solution, 0.03% (w/v)

Weigh 30 mg carbazole (available from ICI Americas, Inc., Wilimington, Delaware 19897 USA) into a 100 ml volumetric flask. Add 50 ml ethylene glycol monomethyl ether and make up to volume with water. Shake the contents to dissolve and add 0.5 ml Renex–30 solution. Keep the reagent in a brown bottle and store in refrigerator. This reagent is stable for four days.

### Sulphuric acid solution, 80% (v/v)

Cautiously, add 1600 ml concentrated sulphuric acid to 400 ml cold water. Add 1.0 ml Renex-30 solution and mix.

### Fructose standard solution a) Stock solution: 1 g/l: Dissolve 100 mg D-fructose in 100 ml water
b) Working standards: 0.02, 0.04, 0.06, 0.08 and 0.10 g/l: pipette 2, 4, 6, 8 and 10 ml of stock solution respectively into 100 ml volumetric flasks and make up to volume with water.

### Equipment

A Technicon AutoAnalyser II (AAII) Continuous-flow Analytical System: is used incorporating a Sampler IV, with a cam to accommodate 20 samples per hour with 3:1 sample-to-wash ratio, a proportioning pump III, a water jacketed coil, 12" dialyser, a heating bath (H" Coil), and a colorimeter equipped with a 15mm flowcell and 560 nm filters, a

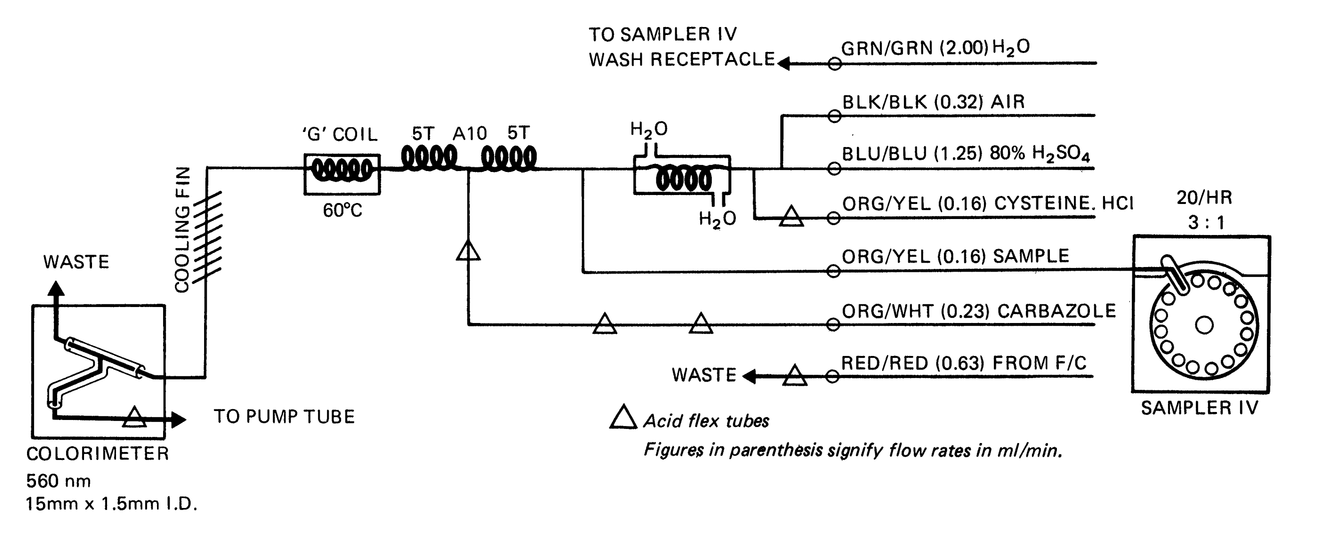

*Figure 1 Flow diagram for the automated analysis of fructose from fermentation media.*

voltage stabiliser. The output is recorded in a recorder. All glassware and fittings used were (AAII)models.

A flow diagram showing the design of the automated modules for the analysis of fructose is shown in Figure 1.

The Sampler IV is set to sample at the rate of 20 samples per hour and heating bath allowed to stabilise at 60°C. The sample is pumped and allowed to mix with cooled and air segmented sulphuric-acid solution containing cysteine hydrochloride. The mixture is mixed in a 5 turn coil and reacted on-line with carbazole. The mixture is allowed to enter the inlet of the heated coil and on exit, the mixture is partially cooled in a cooling-fin. The reagent stream is fed to the colorimeter and the absorbance of the reagent stream is then measured at 560 nm.

### Sample preparation

Samples containing fructose in the range 10–100 g/l require dilution prior to analysis by the present analytical procedure. Dilution of samples may be carried out manually or by online using a dialyser module. A typical flow diagram incorporating a 12" dialyser for the on-line dilution of samples is shown in Figure 2. Samples are aspirated and mixed with air segmented sodium chloride solution containing Renex-30. The mixture is mixed in a 10 turn coil and passes the dialyser. The recipient stream contains sodium chloride and Renex-30 solution. A portion of the stream containing dialysed fructose is resampled and aspirated into the main analytical system for analysis in the conventional manner.

### Sample analysis

Prior to analysis the reagent lines and sample line are placed in water containing a few drops of Renex-30 solution and the system pumped through. The recorder chart drive is switched on. The colorimeter is switched to the direct position and standard calibration knob set to 1.0. Using the aperture control knob on the colorimeter, the recorder pen is adjusted to zero. The reagent lines are then placed in their respective containers except the sample line which is aspirating water. After the system has equilibrated, the baseline is set to zero. Fructose standard (0.08 g/l) is then continuously sampled and the peak height is adjusted to read 80 chart divisions. The standard calibration knob on colorimeter is used for this purpose. Standards and samples are then run into the desired sequence and absorbance of the solution, which is proportional to the concentration of fructose in solution is recorded on the chart. The amount of fructose present in the sample is calculated directly from the recorder chart value.

### Methodology notes

1. Multiple working standards are used simply to establish the linearity of the method. For day-to-day operation, a 0.06 g/l standard is recommended for instrument calibration.
2. If steady state becomes noisy, the acidflex pump tubings should be checked and replacements fitted if required.
3. The absorbance reading produced by the reagents should be about 0.06 above the level produced when water is pumped at the baseline. If a reagent absorbance above 0.06 is observed, both of the carbazole and cysteine hydrochloride reagents must be freshly prepared.
4. Alternate ranges may be obtained using the colorimeter control settings.
5. A sample blank reading can be obtained by replacing carbazole reagents with water.

### Results and discussion

Figure 3 illustrates typical calibration curves for fructose standards with manual and automated procedures. The slopes for the two curves are different, indicating a difference in sensitivity. Variations in experimental conditions used for these methodologies may account for this discrepancy. The reproducibility of the results (precision of the method) was established by replicate analyses of the same sample on the same day as well as over extended periods (2 days) of time. The coefficient of variation was found to be ± 1.11%. The total elapsed time from introduction of sample until appearance of a readout on the recorder chart was 6 minutes.

In Table 1, a comparison has been made between the results obtained by the automated procedure and results obtained manually for seven fermentation samples. The results agree fairly well. The manual method includes several steps which might introduce errors so the comparison must be made with some reservation. Recovery of fructose was measured by adding a known amount of fructose to the preanalysed real samples. The recoveries found ranged from 95% to 101%.

The fundamental chemical reactions involved in the procedure described here are similar to the reaction sequences originally described by Dische [9]. The reaction conditions

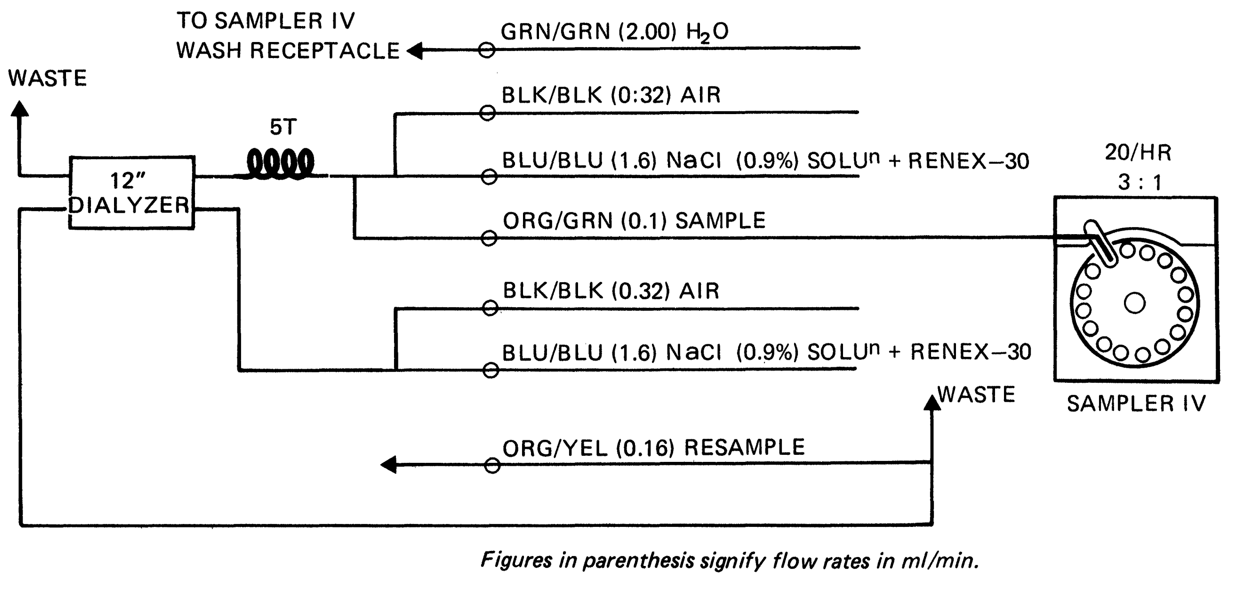

*Figures in parenthesis signify flow rates in ml/min.*

*Figure 2 Flow scheme for automateddilution of samples containing fructose in the range of 25–100 mg/ml.*

were selected so that optimum colour development from fructose takes place at milder experimental conditions to avoid interfluence caused by the presence of other sugars and interfering substances [9]. For optimum colour formation at 60°C, the following amounts of reagent were required: cysteine hydrochloride (2%, w/v), carbazole (30 mg/100 ml) and sulphuric acid (80%, v/v).

Table 2 shows the absorbance characteristics of standard fructose solution (0.04 g/l) when both the amount of reagents and reaction temperatures are varied. While varying the respective amounts of cysteine hydrochloride, carbazole and sulphuric acid, and change in reaction temperatures, the amounts of other reagents and the reaction temperature (60°C) needed for optimum colour formation were used. Increase in reaction temperatures from 55°C to 80°C produced linear increase in colour absorbance. However,

**Table 1. Comparison of the amount of fructose obtained by analysing samples with both manual and improved automated procedures.**

| Sample | Amount of fructose found (micrograms) | |
|---|---|---|
| | Manual (A) | Automated (A) |
| 1 | 20.8 | 19.5 |
| 2 | 13.5 | 13.0 |
| 3 | 5.5 | 6.0 |
| 4 | 5.2 | 4.5 |
| 5 | 12.0 | 14.0 |
| 6 | 12.9 | 13.0 |
| 7 | 9.3 | 9.5 |

A – Samples are analysed in duplicate. Sample readings corrected for blanks.

**Table 2. Absorbance characteristics of D–fructose solution (0.04 mg/ml) with varying amounts of reagents and the increase in reaction temperature from 55°C to 90°C.**

| Reagent | Amount of reagent used (mg/ml) | Observed optical density |
|---|---|---|
| Cysteine hydrochloride | 1.2 | 0.16 |
| | 2.5 | 0.24 |
| | 5.0 | 0.38 |
| | 10.0 | 0.40 |
| | 20.0 | 0.42 |
| | 40.0 | 0.42 |
| | 80.0 | 0.42 |
| | 160.0 (a) | 0.39 |
| | 250.0 | 0.35 |
| | 300.0 | 0.32 |
| Carbazole | 0.05 | 0.22 |
| | 0.10 | 0.19 |
| | 0.20 | 0.33 |
| | 0.30 | 0.35 |
| | 0.60 (b) | 0.36 |
| Concentrated $H_2SO_4$ | (ML/ML) | |
| | 0.6 | 0.10 |
| | 0.7 | 0.35 |
| | 0.8 | 0.43 |
| | 0.9 (c) | 0.91 |
| Temperature | (°C) | |
| | 55 | 0.30 |
| | 60 | 0.51 |
| | 70 | 0.62 |
| | 80 | 0.75 |
| | 90 (d) | 0.92 |

(a) Baseline begins to drift downward. Decrease in sensitivity observed.
(b) Reagent precipitated in transmission tubing. Baseline drifted upward.
(c) Erratic baseline and noisy peaks are obtained.
(d) Deterioration in bubble patterns and noise in peaks are observed.

reaction temperatures above 60°C produced an erratic baseline and noisy peaks. At higher temperature (above 60°C) the potential interferences from glucose present in real samples was also increased.

No colour development at 60°C occurred when either carbazole or cysteine is replaced with water. If 2-mercaptoethanol is substituted for cysteine hydrochloride, a similar colour intensity is produced. Since, fructose under certain experimental conditions forms a reactive glucosone derivative, the reaction steps by which cysteine reacted with fructose may be similar to these for the reaction between 2-mercaptoethanol and o-phthalaldehyde [15].

When ethanol was used as a solvent for carbazole [13] the bubble patterns generated spiked peaks, especially at temperatures above 60°C. Ethylene glycol monomethyl ether is a good substitute for ethanol and eliminates the breakup of bubbles and noise from peaks.

Attempts to use cysteine hydrochloride and carbazole as a combined reagent in aqueous ethylene glycol monomethyl ether were not successful. Carbazole in 80% sulphuric acid followed by the addition of cysteine hydrochloride and then carbazole also produced low sensitivity and noisy peaks. The order of addition of reagents is critical for both the optimum colour formation and for the smooth operation of the analytical system. These observations are incorporated in the flow diagram (Figure 1) developed for fructose analysis.

A typical substrate for enzymatic conversion of glucose into fructose may contain glucose, maleic acid, calcium chloride, magnesium sulphate, potassium chloride and sodium hydroxide. To analyse the fructose content, these substrate samples are neutralised to a pH 6.5 with sodium hydroxide or with hydrochloric acid.

A standard fructose solution (0.05 g/l) was spiked with increasing amounts of the selected interfering substances and analysed. No significant interferences were observed when glucose, maleic acid, maltose, and calcium chloride were present in amounts up to 0.6, 1.0, 0.25 and 0.47 g/l, respectively.

The procedure developed has been shown to work well under routine conditions in the control of isomerase-production facilities. The manual cysteine-carbazole-sulphuric

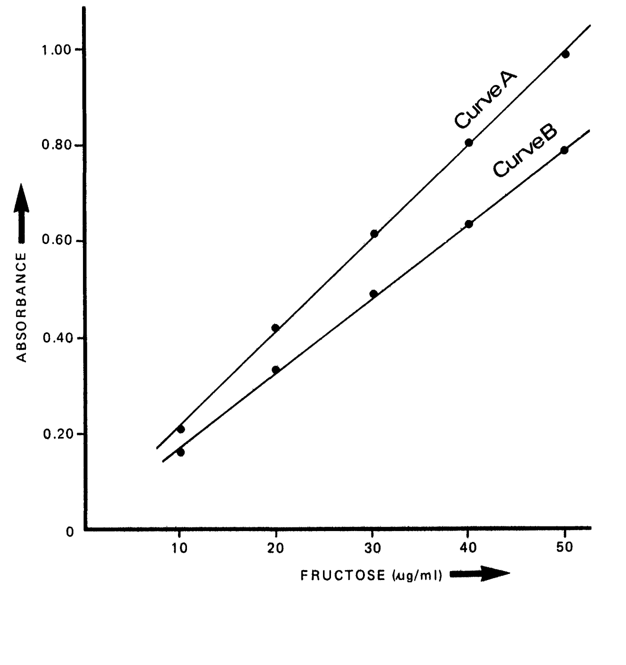

*Figure 3 Calibration curves for fructose standard; curve A is obtained by manual procedure; curve B is obtained by improved automated procedure.*

acid method has been accepted as a satisfactory procedure for analysing fructose as a measure of enzyme activity [12], therefore the present improved automated procedure presented here should be similarly acceptable for measuring fructose from a wide variety of sample matrices.

REFERENCES

[1] Wardrip, E. K., *Food Technol. (London)*, 1971 **25**, 5, 47.
[2] Tsumura, N. and Sato, T., *Agri. Biol. Chem. (Japan)*, 1965 **29**, 2, 1123.
[3] Takasaki, Y., *Agri. Biol. Chem. (Japan)*, 1966 **30**, 2, 1247.
[4] Jermyn, M. A., *Anal. Biochem.*, 1975 **68**, 332.
[5] Halhoul, M. N. and Kleinberg, I., *Anal. Biochem.*, 1972 **50**, 337.
[6] Nixon, D. A., *Clin. Chim. Acta*, 1969 **26**, 167.
[7] Shahidullah, M. and Khorasani, S. S. M. A., *Anal. Chim. Acta*, 1972 **61**, 317.
[8] Zender, R. and Falbriard, A., *Clin. Chim. Acta.*, 1966 **13**, 246.
[9] Dische, Z. and Borenfreund, *J. Biol. Chem.*, 1951 **192**, 583.
[10] Dische, Z., *Methods in Carbohydrate Chemistry*, 1962 **1**, 481.
[11] Cadmus, M. C. and Strandburg, G. W., *Anal. Biochem.*, 1968 **26**, 3, 484.
[12] Lloyd, N. E., Khaleeluddin, K. and Lamm, W. R., *Cereal Chemistry*, 1972 **49**, 544.
[13] Nakamura, M., *Agri. Biol. Chem.*, 1968 **32**, 6, 701.
[14] Kainuma, K., Tadokoro, K., Sugawara, K. and Suzukis, S., *Dempun Kogyo Gakkaishi*, 1967, **15**, 1, 10, *Chem. Abstr.* 1968 **69**, 83953.
[15] Simons, S. S. and Johnson, D. F., *J. Org. Chem.* 1978, **43**, 14, 2886.

### Meeting Reports

### ECCLS discussion group meeting on instrument testing

At a recent meeting of the newly formed European Committee for Clinical Laboratory Standards (ECCLS) several *ad hoc* groups met to propose policies which this Committee might pursue. Groups met to cover the subjects of labelling, specimen collection, materials for quality control and quality control procedures. Reports from all groups are available to ECCLS Members from Irene Batty, ECCLS Executive Director, Wellcome Research Laboratories, Langley Court, Beckenham, Kent, England. The report from the Instrument Testing Group is reproduced below. It is emphasised that the proposals do not necessarily represent the policy of the ECCLS Board.

The Group was instructed to propose a policy which the Committee might pursue in the important area of instrument testing. Those present represented the three major participating sectors in the membership of ECCLS with three members from government, three from industry and seven from the professions. *

During a wide-ranging discussion the following conclusions were reached:

1. Work in the area of instrument evaluation should be given a high priority in the initial programme for the ECCLS. Evaluation protocols should be produced as quickly as possible and a scheme should be inaugurated for organising instrument evaluations to cover the member countries of ECCLS.
2. For the above purposes a Working Group should be formed with a membership coverage similar to that of the present *ad hoc* group but including a professional statistician.
3. The present replication of effort in this area is extremely wasteful in time and money both for industry and the profession and the resulting evaluations are all too often of a superficial nature.
4. Evaluations and any recommendations should be done and made by potential users. Specialists should look into certain specific areas, ie a photometer or a syringe system if present. Testing should be done to a parametric standard, ie the protocol should describe how the test is to be carried out and should not lay down limits.
5. Any previous testing done by the US National Committee for Clinical Laboratory Standards (NCCLS) or another organisation should be taken into account in arriving at conclusions. If instruments have been in use in laboratories prior to the testing scheme commencing, user experience should be canvassed.
6. A general protocol should be produced to cover all types of instruments for all specialties within pathology. This would then be divided into sections including for example (a) electrical safety (this must comply with the rules in individual countries), (b) haematology, (c) clinical chemistry. Clinical chemistry and haematology would then be broken down for various types of instruments, ie for clinical chemistry – flame photometers and colorimeters. Several instruments (eg colorimeters) are common.
7. Each instrument will inevitably require a slightly different approach and the ECCLS must therefore determine a revised protocol applicable to a particular instrument before testing is commenced.
8. Special arrangements should be made for instruments specifically constructed for use in doctors' surgeries, wards, etc.
9. Before every evaluation a contract should be carefully drawn up between the producer and the testing organisation. The evaluation process should be done as quickly as possible. All instruments tested must be production models. No prototypes should be tested in the proposed scheme. Testing should always be done against a carefully prepared protocol and there should therefore be no chance of litigation by the producer.
10. The overall arrangements must include a means whereby the producer can comment upon the final report, and his comments should be published with the report.
11. The ECCLS Group would ideally pick three routine laboratories in at least two different countries where testing should take place simultaneously. In each laboratory at least three different technicians should be involved with operating the instrument on their own if possible. It may not be practical to install three large instruments at one time and arrangements may be modified to cover this difficulty.

### Implementation

Professor Haeckel (Hannover) together with a selected group in Germany has produced a protocol at the instigation of the IFCC Expert Panel on Instrumentation. The German Group is currently using the protocol in an instrument programme and in the light of experience so gained, it will be modified and subsequently tested again jointly with a French Group. This protocol could form the basis of an ECCLS document. It is hoped that a final version should be available in the summer of 1980 and thereafter no time should be lost in the ECCLS taking it up for use. The IFCC Expert Panel would endeavour to obtain full international acceptance

**Membership of Instrument Testing Group:-*
*P Bonini, Karl-Georg von Boroviczeny, Christian Collombel, J Dautlick, E J Dixon, F Esser, T Gerritsen, J Bierens de Haan, W Jansen, G Merry, F L Mitchell (Chairman), M Roth, D B Slade.*

